# Supplementary material for: High rate of partner violence during pregnancy in eastern Ethiopia: Findings from a facility-based study
Source: PLoS One. 2020 Jun 4;15(6):e0233907. doi: 10.1371/journal.pone.0233907 (PMC7272015; doi:10.1371/journal.pone.0233907)
Supplement: Attachment — Submitted filename: Response to reviewers.docx [file pone.0233907.s001.docx]

**Response to Editor and reviewers**

Research title: High rate of partner violence during pregnancy in eastern Ethiopia: Findings from a facility-based study.

**Dear Editors**

We appreciate your effort in considering our article for further review. We also thank the reviewers for their genuine comments regarding the modifications needed to our paper. We have made the required changes to the manuscript and have summarised our responses to each reviewer below.

| **Response to Editor’s comments** | | |
| --- | --- | --- |
| **No** | **Comments** | **Response** |
| 1 | Please include additional information regarding the survey or questionnaire used in the study and ensure that you have provided sufficient details that others could replicate the analyses. For instance, if you developed a questionnaire as part of this study and it is not under a copyright more restrictive than CC-BY, please include a copy, in both the original language and English, as Supporting Information | The questionnaire we used to collect this data was obtained from WHO Multi-country VAW study. This questionnaire will be submitted and published in University Newcastle online thesis repository as part of PhD thesis. However, we extracted specific items that were used to measure the outcome variable and included them in the methodology section in the current manuscript as a table (Table 1). |
| 2 | Please describe how verbal consent was documented and witnessed | To address this, the following sentences were added to information under Ethical clearance *“First, the interviewer read out participant’s information sheet slowly. Information regarding purpose of the study, risk and benefit of participating, measure taken to protect participants’ privacy and their choice to participate in the study were explained. Then, they were given adequate time to ask questions to ensure they had a clear understanding of the study. After their questions were addressed, their consent was sought by asking the participant to respond affirmatively to a series of statements. The interviewer signed and dated the consent form”.* |
| 3 | Please refrain from stating p values as 0.000, either use the exact number or the format p<0.001. | Comment accepted and corrected in the current manuscript. |
| 4 | Your ethics statement must appear in the Methods section of your manuscript. If your ethics statement is written in any section besides the Methods, please move it to the Methods section and delete it from any other section. Please also ensure that your ethics statement is included in your manuscript, as the ethics section of your online submission will not be published alongside your manuscript. | Our ethical clearance statement appears under the methodology section. In the current manuscript version, the approval numbers for respective Ethical clearance have been inserted. |

| **Responses for Reviewer 1** | | |
| --- | --- | --- |
| **No** | **Authors’ Response** | **Reviewer’s Comments** |
| 1 | **General comments**  The authors justify the need for this study because the limited information on this problem in this specific region. However, they mention the existence of other studies in Ethiopia. Could the authors indicate what differentiates this region from those already been studied? In other words, why study this specific region is relevant? | This region is different from most of the regions in Ethiopia. Some of these differences are described below:   A) Residency: The Ethiopian population is predominantly rural (86%). However, Harari region is the only region in Ethiopia where the majority of its population live in urban locations (55%) as described under the study setting in the manuscript. Residency is one of the important factors that can affect IPV[1]. B) Religion: about one third of the Ethiopian population are Muslim but in Harari region more than two thirds of the population are Muslim. Differences in religion might contribute to IPV as the religion might be misused to justify IPV. For example, in some Muslim countries, selective quotes from the Quran may be used to ‘prove’ that it is allowable for a man to ‘beat’ his wife [2] which might embolden the perpetrator C) Substance use: Although about 16% of males in Ethiopia use Khat, in Eastern Ethiopia (including Harari region) a relatively higher proportion of the population use Khat, Evidence has indicated more than 32% of women and 73% of males use Khat [3]. Using Khat and other drugs were associated with an increased risk of IPV [4, 5]. In the Ethiopian context, regions are semi-autonomous and they have their own health bureau that leads interventions related to health in the region. Therefore, region based information regarding IPV is crucial to make intervention that reduce IPV among pregnant women in the region.   This detail is included in the introduction part of the manuscript as follows ”*Harari region has the highest proportion of population who reside in urban (55%) than any other regions in Ethiopia and the majority of its population are Muslim (69%) [6]. Both residency [1] and religion [2] are important factors that can affect the prevalence of IPV. For example, evidence indicated that in some Muslim countries, selective quotes from the Quran may be used to ‘prove’ that it is allowable for a man to ‘beat’ his wife [2] indicating the role of religion in promoting social control. Therefore, the generalizability of the study that was conducted in one part of the country might not provide the true prevalence of IPV in the other region as IPV might be affected by within-country differences in cultures and societal norms [7] since Ethiopia is a multi-cultural state”* |
| 2 | **Abstract** Please, correct the sentence: “Variables with a p-value of ≤0.05 were considered to have an association with intimate partner violence during pregnancy”. | This sentence is corrected as follows “*Variables with a p-value of ≤0.05 were considered to have a significant association with intimate partner violence during pregnancy”* |
| 3 | **Introduction** After indicating that Ethiopia is a multi-cultural state, the authors describe dowry as a practice that increases the risk of IPV for women. They also describe two types of dowry with potentially different results for women. However, it is not clear how this practice is in the Harari region. | The dowry practice in Harari region is similar to Ethiopia. In Harari region, the dowry is given to the bride’s family as cash or cattle. This statement is included in the current version of the manuscript as follows*: “The relationship between dowry payment and partner violence has long been established [8]. Unlike countries in Asia that offer a dowry to the groom’s family [9], in Ethiopia a dowry of cash or cattle is given to the bride’s family before the marriage [10]; this is the practice in Harari region.”* |
| 4 | **Method** It is not clear if data collection was completed before or after births. Hence, it is not obvious what the authors mean when they say “the most recent pregnancy”? Is it the current pregnancy? In page 13, it seems that some interviews were completed before and other after births. Please, clarify this. | Under the participant section, we mentioned, “*All women who gave birth in the public hospitals found in the Harari region were included in the eligible study population*”. This is to indicate that data collection was conducted on women who gave birth in the hospitals (during postpartum period), not on pregnant women. To avoid confusion, under the “participants” sub section we added this sentences “*Participants were recruited following the birth of their baby in the hospitals*”. Further, we have replaced the “*current pregnancy*” with the “*most recent pregnancy*” in the current manuscript.” We used the most recent pregnancy to indicate that women were asked whether their experienced IPV during their last pregnancy. |
| 5 | It would be necessary to know the format of the questions posed and some examples of them. | This study is part of larger PhD study to investigate IPV in the region. Although the questionnaire has multiple sections that will be submitted to the University for online publication after the completion of the study, we extracted the part that addressed IPV and summarized this in the table in the methodology section. The questionnaire we have used is adapted for the Ethiopian context, as Ethiopia is one of the countries that was included in WHO multi country study that was conducted in 2005. Therefore, we used the same tool that are commonly being used in Ethiopia by researchers [11-13]. |
| 6 | **Statistical Analysis** This section needs a more detailed description of the steps followed in the analysis. For instance, could you indicate how you set the levels of the outcome to compute the logistic regressions? (i.e., was it used a "yes" or "no" binary category?). Could you indicate the method used to introduce the predictor variables into the analyses? Moreover, it is not clear how and why the authors computed a binary regression and, subsequently, a multiple logistic regression. How did they change an outcome with two levels to more than two levels? The authors need to explain this better? | Comment accepted and the following modification was made.  The detail about items used to measure each aspect of IPV was provided in the manuscript under methodology by using table. As it appears on the Table 1, physical IPV was assessed by 6 items, psychological IPV was assessed by 4 items while sexual IPV was assessed by 3 items. All the questions asked were ‘Yes’ or ‘No’ question. Women who reported ‘Yes” for at least one of the physical IPV items were considered to have experienced physical IPV. Similarly, women who responded ‘Yes’ for at least one of the emotional IPV measurement items were considered to have experienced emotional IPV while those who replied “yes’ for at least one of the three sexual IPV items were considered to have Sexual IPV. For overall IPV, women who reported experiencing any types of IPV (whether physical, emotional or sexual) were considered to have IPV during the most recent pregnancy. This overall IPV was used as an outcome and it is categorical variable with only two possible outcomes (Yes and No for IPV).  Regarding analytical statistics, since the outcome variable is a categorical variable we can do both binary logistic regression and multiple logistic regression. In binary logistic regression, we entered variables one by one in the logistic regression model to see its individual associations. However, to conduct multiple logistic regression, we entered more than one variable to get the adjusted relationship between IPV and other explanatory variables. The predictor variables were selected based on the findings of relevant literature as well as statistical significance |
| 7 | **Discussion** The discussion outlines the findings and situates these alongside the reviewed research. The paper also refers to some study limitations. However, the authors could also refer to the consequences of IPV on the health of women and their children as a means to demand more intensely a change in social norms that condone violence. In a social context with such a high acceptance of IPV, hospitals can do more than point out that violence exists. They can focus on the seriousness of the consequences that violence entails in order to start raising awareness. For the future, the authors could find interesting to analyze the consequences of IPV on the health of women and neonates. It is strange that no woman accepted the help offered by the violence she was suffering. Authors should try to explain this. | This is very important point and we made modifications to address these comments. We agree that hospitals could do more than indicating that violence exists. However, to support awareness creation it is important to know the impact of IPV on adverse pregnancy outcomes using local studies. Therefore, we included recommendations that indicate the importance conducting studies to investigate the consequence of IPV. This is described in the conclusion as follows; “….*to support this awareness creation using local evidence, further research that investigate the consequences of IPV on pregnancy outcomes in the Ethiopian context are needed*” As mentioned earlier, this is study is part of larger study that include investigating the consequence of IPV. We have aim that investigated the consequence of IPV and the manuscript that was written on the consequence of IPV (adverse pregnancy outcomes) is currently under review. In that manuscript, broader finding-based recommendation that are critically important to address IPV were included.  Furthermore, the explanation regarding why woman do not accept offers was described in relation to attitudes towards IPV in the discussion. It was presented as follows: “… *It is important to note that none of the women who experienced IPV in the current study took up the offer of support. Although further study is needed to understand why abused women may not want to get support, this might indicate an overall tolerance of violence within the community and its role in fostering a conducive environment for abusers [14]”.* |

| **Responses for Reviewer 2** | | |
| --- | --- | --- |
| **No** | **Authors’ Response** | **Reviewer’s Comments** |
| 1 | **General comment**  Authors made an exploratory analysis. I personally think that a hypothesis-based analysis would make more sense. In my opinion, the current approach is a little bit confusing. | Although we clearly did not describe all potential variables that were investigated in relation to IPV in the form of a null hypothesis and an alternative hypothesis, our analysis still tested the significance of associations between variables. As was described in Methodology section, we have used previous literature to identify variables potentially associated with IPV and then testing those associations.  In the current manuscript, to clearly indicate this , we included the research question as follows “*In other word, the study attempted to address the following question: ‘is there an association between IPV and a given set of sociodemographic factors, marital and behavioural factors, reproductive and physical health-related factors, attitude towards IPV and partner controlling behaviour*?. |
| 2 | **Abstract**  Please, provide the explanation for AOR and CI. | We have put the explanation for both AOR and CI as a footnote in Table 6 in the current version of the manuscript. |
| 3 | **Abstract**  The second sentence of the conclusion is not necessarily drawn from the results.  ”. | We corrected the conclusion as per the reviewer’s recommendation as follows: “*The prevalence of intimate partner violence during pregnancy was found to be high. Pregnancy monitoring programs, which can detect and intervene with regard to partner’s controlling behaviors and women’s perception regarding justification of IPV, especially in those women with an unplanned pregnancy, could help to reduce IPV. Further, changing social norms that condone violence through advocacy and awareness creation might help in preventing partner violence”* |
| 4 | Introduction: The first paragraph does not seem necessary to me.  . | The first paragraph talks about the definition of IPV used in the current manuscript. We felt that this is important to introduce the reader with the concept of IPV, how it was defined and which definition is used in this manuscript. We have therefore opted to leave the paragraph. |
| 5 | The paragraph on page 4 that begins with “The relationship between dowry payment…” should be revised. From my point of view, too much importance is given to dowry as a explanatory factor, but then not throughout the text. Perhaps it is only necessary to comment that some factors related to IPV during pregnancy have been identified and give some examples. In the current version, it is not known whether the factors cited are related to IPV or IPV during pregnancy. | It is true that although we mentioned other factors that are associated with IPV, we gave more emphasis to dowry practices. And as per Reviewer 1, comment, we have clarified the importance of dowry in Ethiopia. We did this to introduce the reader to dowry practices in the Ethiopian context because we have used this variable for calculating sample size. |
| 6 | Methods: Comment 5:The first section could be entitled "Study design" and include soon the mention to "cross-sectional study". | Comment accepted and corrected in the current version as follows: “*Study design: cross-sectional survey of women attending public hospitals found in the Harari region was conducted from 25 November 2018 to 28 April 2019”.* |
| 7 | The dowry payment exposure was used to calculate the sample size, but it is not known why and, in any case, an estimate of its magnitude is lacked. | This study is part of larger study that investigated the magnitude, associated factors, attitudes and outcomes of IPV during pregnancy in eastern Ethiopia. The sample size that addresses all aims was calculated by using two scenarios (the single proportion population formula and associated factors). Obtained from previous literature, the prevalence of IPV, and proportion of women with attitude supportive of IPV were used to calculate sample size and the largest sample size (from the two proportion) was identified. Similarly, for associated factors, we have used variable that gave us larger sample size considering associated factors of IPV, factors associated with having a perception that justify IPV and pregnancy outcomes. From overall sample size calculated including sample size calculated by using single population proportion formula, the sample size calculated using dowry practice gave us largest sample size. Therefore we used it for calculating overall sample size. As we never used the proportion of dowry practices to calculate sample size, we do not think that it is important to mention the prevalence of dowry practices in this method. That is why we described dowry practice as a factor rather than describing its proportion. |
| 8 | How many items does the questionnaire have? How many for each type of violence? Could you provide details of its validity? | Overall, there are 13 items used to assess IPV. Physical IPV was assessed by 6 items, psychological IPV was measured by 4 items while sexual IPV was measured by 3 items. This questionnaire was adapted from WHO multi-country study questionnaire. WHO conducted study to provide the overall global prevalence of violence against women in 2005, and Ethiopia was one of the countries that was included in this global study. The overall questionnaire was adapted to the Ethiopian context by removing some of the items that could not be applied in Ethiopia context, as described in the Methodology section of WHO study. These items included questions that were designed to addresses the impact of violence on mental health. However, specific items that measured IPV were found to be valid in the Ethiopian context and none of them were removed. This questionnaire has been widely used by different researchers in Ethiopia to study IPV [11-13].  For clarity, we have included the items used to measure IPV in the manuscript in Methodology (Table 1). |
| 9 | Generally, there are too many variables. I believe that a selection could be made with the most relevant according to the scientific literature and the most explanatory according to the preliminary analyses. | We agree with the reviewer. As mentioned previously, we have collected many variables that are intended to address all outcomes we were looking to address in the wider study. Although some of them can provide further descriptions about the population, it is not necessary to include all of them in the current manuscript. Therefore, we removed some of the details that were addressed in other manuscripts. For example, we removed some of the variables that are related to pregnancy outcome as we have another manuscript dedicated to this.  Specific to the variables entered in to the multiple logistic regression, first we did not included all variables in the binary logistic regression. It is true that model with more variables presents less statistical power, which can lead to wrong conclusion. To avoid this, we first fitted binary logistic regression using relevant variables that were only supported by previous literature. Then, we observed its level of significant in bivariate analysis and variable with a p-value of ≤0.05 were transported to the multiple logistic regression. In addition, there were variables that were significant but were not transferred to multiple logistic regression (E.g. gravidity). This decision was made because parity and gravidity are similar concepts and the variables were highly correlated. Therefore, we excluded gravidity from multivariate analysis. Overall, we have used a combination of statistical significance and relevant literature to determine variables that were entered into the logistic regression model.  To address this, the methodology section of the manuscript was edited as follow: “*three logistic regression models were fitted to investigate the associated factors of IPV. In the first model (Model 1), binary logistic regression was conducted to determine associations between IPV and potential explanatory variables. All potential explanatory variables that were fitted in binary logistic regression were obtained from relevant literature. In the second model (Model 2), variables (excluding attitude towards IPV and partner controlling behaviour) that demonstrated a significant association (with a p-value of ≤0.05) in bivariate analysis were exported to a multiple logistic regression model for multivariate analysis”.* |
| 10 | Results: I would prefer to see the first tables and figures with the total percentages (like now), but also the percentages according to having suffered from IPV (and not). That is, three columns, one with total n (%), one with n (%) for those without IPV, and another column for those with IPV. A fourth column can be displayed with p-values. | Comment accepted and appropriate changes made. |
| 11 | Figures mixing absolute and relative values don't make much sense. | Comment accepted and corrected. The figures were described by percentages only. |
| 12 | In Table 5, mixing the variables that reflect the partner’s control and the attitude towards IPV with the other variables is confusing for me. There may be overlapping issues and it does not help to understand the phenomenon. These two variables could be used to make strata, and study the effect of the other variables on them. In sum, these variables could be on the causal pathway.  . | Comment accepted and modified as per reviewer’s recommendation.  In this study, we were not interested in conducting pathway analysis. Rather, we included partner control and women’s attitudes towards IPV with other variables considering that all variables can independently contribute for IPV. This approach was used in a number of previous studies (Black, E., Worth, H., Clarke, S. *et al.* Prevalence and correlates of intimate partner violence against women in conflict affected northern Uganda: a cross-sectional study. *Confl Health* **13,** 35 (2019). <https://doi.org/10.1186/s13031-019-0219-8>, Tlapek, S. M. (2015) ‘Women’s Status and Intimate Partner Violence in the Democratic Republic of Congo’, *Journal of Interpersonal Violence*, 30(14), pp. 2526–2540. doi: [10.1177/0886260514553118](https://doi.org/10.1177/0886260514553118), Shai N, Pradhan GD, Chirwa E, Shrestha R, Adhikari A, Kerr-Wilson A (2019) Factors associated with IPV victimisation of women and perpetration by men in migrant communities of Nepal. PLoS ONE 14(7): e0210258. <https://doi.org/10.1371/journal.pone.0210258>). However, in the current version of the manuscript, we constructed three different models. The first model contains bivariate analysis of all variables, while the second model contain all variables found to have significant associations in bivariate analysis, excluding attitude towards IPV and controlling behaviour. The last (third) model included all variables found to have significant associations, including attitude towards IPV and partner controlling behaviour. Within both multivariate models, there was no big change in terms of effect size though duration of marriage that had become insignificant in model 2 became significant in Model 3. To investigate whether attitude towards IPV and partner controlling behaviour moderates the duration of marriage, the interaction analysis was done but it was not significant. Therefore, we did not include this interaction term in the model, as it was not significant. The discussion was made based on the final model (Model 3). |
| 13 | **Conclusions:** Some of them are not based on results. Some are general comments, probably true, but not followed directly from this study. From this work it can be concluded, for instance, that a pregnancy monitoring program is necessary, which can detect partner’s control behaviors and women justification of IPV, especially in those women with an unplanned pregnancy.  .” | Comment accepted and the conclusion is modified based on reviewer’s suggestion. The conclusion was re-stated as follows: *“The prevalence of IPV during pregnancy is high; two in five women experienced IPV during their most recent pregnancy.* *Pregnancy monitoring programs, which can detect and intervene with regard to partner’s controlling behaviors and women’s perception regarding justification of IPV, especially in those women with an unplanned pregnancy, could help to reduce IPV. Further, changing social norms that condone violence through advocacy and awareness creation might help in preventing partner violence [15, 16]. To support this awareness creation using local evidence, further researches that investigate the consequence of IPV on pregnancy outcomes are needed. Law enforcement agencies should strictly implement legislation that prevents violence against women, which in the long term will aid in creating societies that do not tolerate violence [17]*. |

**References**

1.	Naved RT, Persson LA: **Factors associated with physical spousal abuse of women during pregnancy in Bangladesh**. *Int Fam Plan Perspect* 2008, **34**(2):71-78.

2.	Douki S, Nacef F, Belhadj A, Bouasker A, Ghachem R: **Violence against women in Arab and Islamic countries**. *Archives of women's mental health* 2003, **6**(3):165-171.

3.	CSA [Ethiopia], ICF: **Ethiopia Demographic and Health Survey 2016. ,** . In*.* Addis Ababa, Ethiopia and Rockville, Maryland, USA; 2016.

4.	Kassa ZY, Menale AW: **Physical violence and associated factors during pregnancy in Yirgalem town, South Ethiopia**. *Current Pediatric Research* 2016, **20**(1):37-42.

5.	Lencha B, Ameya G, Baresa G, Minda Z, Ganfure G: **Intimate partner violence and its associated factors among pregnant women in Bale Zone, Southeast Ethiopia: A cross-sectional study**. *PloS one* 2019, **14**(5):e0214962.

6.	Central Statistical Agency: **Population Projection of Ethiopia for All Regions At Wereda Level from 2014 – 2017**. In*.* Edited by Agency FDRoECS. Addis Ababa: Central Statistical Agency 2013.

7.	Linos N, Slopen N, Subramanian SV, Berkman L, Kawachi I: **Influence of Community Social Norms on Spousal Violence: A Population-Based Multilevel Study of Nigerian Women**. *American Journal of Public Health* 2013, **103**(1):148-155.

8.	Batra ND: **Why this growing atrocities on women?** *Yojana* 1988, **32**(9):32-34.

9.	Banerjee PR: **Dowry in 21st-Century India: The Sociocultural Face of Exploitation**. *Trauma, Violence, & Abuse* 2013, **15**(1):34-40.

10.	Regassa M, Mitiku T, Hailu W: **Addooyyee: Girl’s Indigenous Friendship Institution in Oromoo, Ethiopia**. *International Journal of Multicultural and Multireligious Understanding* 2019, **6**(1).

11.	Mohammed BH, Johnston JM, Harwell JI, Yi H, Tsang KWK, Haidar JA: **Intimate partner violence and utilization of maternal health care services in Addis Ababa, Ethiopia**. *BMC health services research* 2017, **17**(1).

12.	Yimer T, Gobena T, Egata G, Mellie H: **Magnitude of Domestic Violence and Associated Factors among Pregnant Women in Hulet Ejju Enessie District, Northwest Ethiopia**. *Advances in Public Health* 2014, **2014**:8.

13.	Abebe Abate B, Admassu Wossen B, Tilahun Degfie T: **Determinants of intimate partner violence during pregnancy among married women in Abay Chomen district, Western Ethiopia: a community based cross sectional study**. *BMC Womens Health* 2016, **16**:16.

14.	Kurz D: **SOCIAL SCIENCE PERSPECTIVES ON WIFE ABUSE:: Current Debates and Future Directions**. *Gender & Society* 1989, **3**(4):489-505.

15.	Wagman JA, Gray RH, Nakyanjo N, McClendon KA, Bonnevie E, Namatovu F, Kigozi G, Kagaayi J, Wawer MJ, Nalugoda F: **Process evaluation of the SHARE intervention for preventing intimate partner violence and HIV infection in Rakai, Uganda**. *Evaluation and program planning* 2018, **67**:129-137.

16.	Rivas C, Ramsay J, Sadowski L, Davidson LL, Dunne D, Eldridge S, Hegarty K, Taft A, Feder G: **Advocacy interventions to reduce or eliminate violence and promote the physical and psychosocial well-being of women who experience intimate partner abuse**. *The Cochrane database of systematic reviews* 2015(12):Cd005043.

17.	World Health Organization: **Violence prevention: The Evidence**. In*.* Geneva: World Health Organisatioon; 2010.
