# Supplementary material for: High rate of partner violence during pregnancy in eastern Ethiopia: Findings from a facility-based study
Source: PLoS One. 2020 Jun 4;15(6):e0233907. doi: 10.1371/journal.pone.0233907 (PMC7272015; doi:10.1371/journal.pone.0233907)
Supplement: Attachment — Submitted filename: Response to reviewers2 .docx [file pone.0233907.s002.docx]

**Response to Editor and reviewers**

Research title: High rate of partner violence during pregnancy in eastern Ethiopia: Findings from a facility-based study.

**Dear Editors**

We appreciate your effort in considering our article for further review. We also thank the reviewers for taking time to review the manuscript again. We have made the required changes to the manuscript and have summarised our responses to Reviewr-2 as follow

| **Responses for Reviewer-2** | | |
| --- | --- | --- |
| **No** | **Comments** | **Response** |
| 1 | Thank you for putting the explanation for both AOR and CI as a footnote in Table 6, but I think that it also be helpful in the abstract. | Comment accepted, both AOR and CI were written in full statement in the current manuscript abstract. |
| 2 | I suggest to add some of your comments. For example: The sample size was calculated using significant factors of partner violence during pregnancy obtained from a previous study conducted in Ethiopia [16]. Dowry payment maximized sample size, therefore we used it as exposure variable for calculations… | Comment accepted. The section is modified as follows: *The sample size was calculated using significant factors of partner violence during pregnancy obtained from a previous study conducted in Ethiopia [16].* ***Dowry payment maximized sample size; therefore, we used it as the exposure variable for calculating sample size considering*** *the following parameters: dowry payment as the exposure variable, power level of 80%, and the ratio of unexposed to exposed of 1.0*…. |
